# A Neurotized Anterolateral Thigh Flap With a Unique Anastomosis to the Gastroepiploic Artery: A Case Report of a Reconstruction of Composite Abdominal Wall Defect

**Published:** 2016-07-15

**Authors:** Edward Hahn, Edward S. Lee, Jonathan D. Keith

**Affiliations:** Department of Surgery, Division of Plastic & Reconstructive Surgery, Rutgers University–New Jersey Medical School, Newark

**Keywords:** desmoid, composite, abdominal wall reconstruction, anterolateral thigh free flap, gastroepiploic artery anastomosis

## DESCRIPTION

A 26-year-old woman presented with symptomatic recurrent desmoid tumor to her anterior abdominal wall. The patient underwent radical resection of the desmoid tumor. She was left with only 30% of her anterior abdominal wall intact, creating a large defect requiring composite reconstruction.

## QUESTIONS

1. **What is a desmoid tumor?**
2. **What are treatment options for desmoid tumor?**
3. **What are surgical options for composite abdominal wall reconstruction?**
4. **What specific considerations should be made when deciding a particular reconstructive surgery?**

## DISCUSSION

Desmoid tumors are derived from the proliferation of spindle-shaped mesenchymal fibroblast-like cells. Desmoid tumors often present as an aggressive fibroproliferative process, with a variable clinical course. Metastatic disease and dedifferentiation into high-grade malignancy are not typical features.^1^ However, local recurrence is a common characteristic of these tumors. The incidence of this tumor is approximately 2.4 to 4.3 new cases per 1,000,000 per year. Median age at diagnosis is 30 years; female to male gender ratio is 2:1. The diagnosis is concluded using clinical, histological, and radiographic data. Histological studies demonstrate the behavior of the tumor to infiltrate deep tissues, such as muscles, and along muscle planes. Magnetic resonance imaging and computed tomography are valuable in assessing the extent of disease. The diagnosis is confirmed by biopsy.

Treatment options for desmoid tumors include surgical excision, radiotherapy, systemic medical treatment, or combinations of these.^1^ Regardless of the particular treatment modality, disease eradication is often difficult, with failure rates reported as 25% to 60% of cases at 5 years.^1^^,^^2^ Consequently, large surgical resections are sometimes necessary to optimize outcome and achieve local disease control.

**Figure 1:**
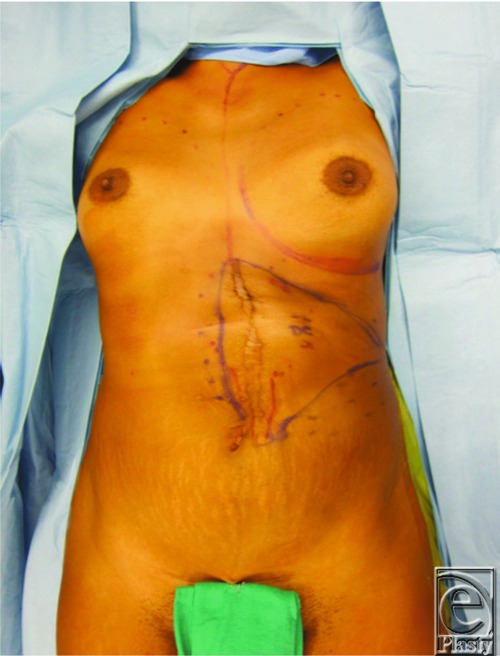
Preoperative.

**Figure 2:**
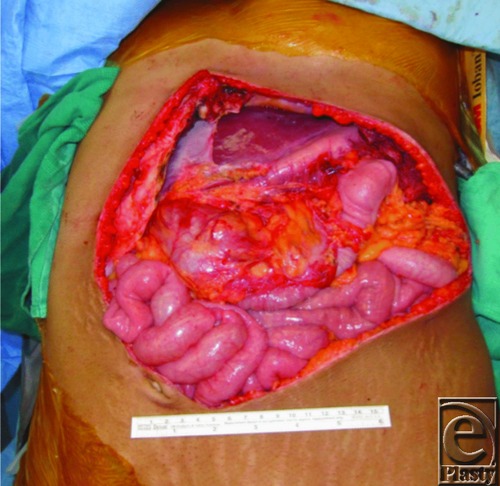
Composite defect after resection.

**Figure 3:**
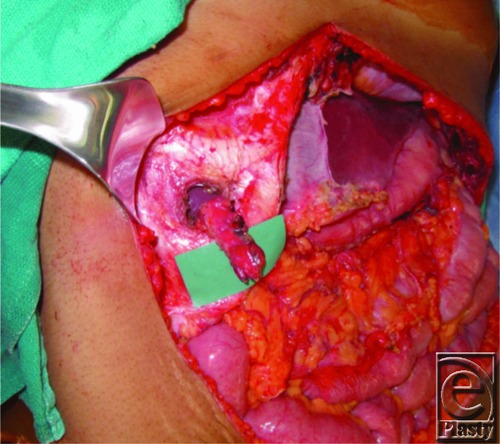
Gastroepiploic artery pedicle passed through an intercostal window.

**Figure 4:**
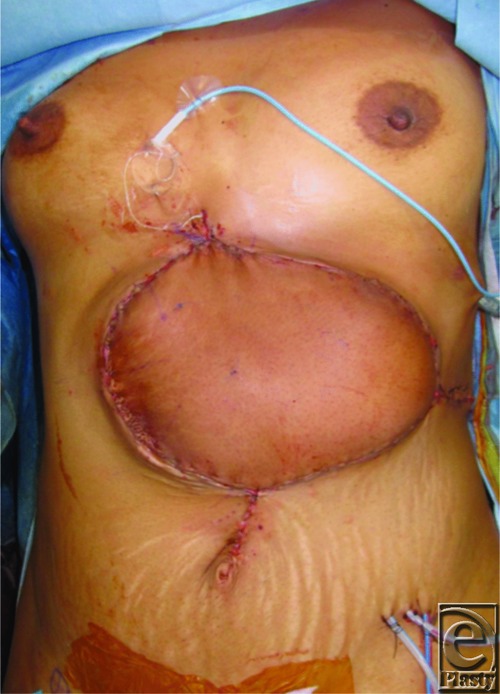
Anterolateral thigh flap inset.

This is a case of a 26-year-old woman who had presented with symptomatic recurrence of desmoid tumor to her anterior abdominal wall (Fig 1). The patient had undergone a radical resection of desmoid tumor from her anterior abdominal wall following recurrence. Portions of the costochondral margin, xiphoid, diaphragm, and pericardium were resected. All soft-tissue layers of approximately 70% of anterior abdominal wall were resected (Fig 2). An anterolateral thigh (ALT) flap measuring 25 x 15 cm was designed on the basis of the descending branch of the lateral femoral circumflex artery and raised with the lateral cutaneous femoral nerve (LCFN). In determining the optimal recipient vessel for the free flap, the internal mammary vessels were considered but would require an additional incision on the chest and décolletage area. To avoid such an unaesthetic incision, we decided to use the right gastroepiploic artery. The gastroepiploic recipient vessels were brought through an intercostal window through the fifth rib space (Fig 3). Anastomosis of the ALT flap vessels to the right gastroepiploic artery and vein was performed. A neurorrhaphy of the fifth intercostal nerve to the LCFN was performed to neurotize the flap. A biological mesh was secured over the abdominal defect for structural support and coverage of the intra-abdominal viscera. The ALT flap was then inset and sutured over the biological mesh (Fig 4). The ALT free flap survived, and the patient was discharged home. At 18 months' follow-up, the patient is without abdominal hernia and has developed sensation in the flap skin paddle.

Composite full-thickness abdominal wall defects continue to be a challenge to the reconstructive surgeon, and special considerations should be made when deciding a reconstructive surgery. The surgeon is required to address the structural, functional, and aesthetic features when considering surgical options. Pedicled ALT flaps are an option for abdominal wall reconstruction. However, the surgeon needs to consider that the pedicled variant is restricted by the pedicle length and therefore is more suitable for reconstruction of the lower abdominal quadrants.^3^ An ALT free flap is often an ideal surgical option.^4^^,^^5^ This case describes a surgical option for large composite abdominal wall reconstruction using an ALT free flap with anastomosis to the gastroepiploic vessels whereby the vascular pedicle was presented through an intercostal window. The intercostal window avoided creating an otherwise necessary hole in the mesh for the flap pedicle. Such a violation of the mesh would create a defect and, consequently, increase the risk of visceral herniation through the mesh. Furthermore, the neurorrhaphy of the fifth intercostal nerve to the lateral cutaneous femoral nerve successfully provided sensation to the flap skin paddle that is acting as the tactile surface of the abdominal wall. The neurotized sensate ALT flap with anastomosis to the gastroepiploic vessels reconstructed the composite abdominal wall defect with structural, functional, and aesthetic success. A successfully neurotized sensate ALT free flap for abdominal wall reconstruction as described in this article has not yet been reported in the literature. Also, this interesting case may serve as “proof of concept” for composite abdominal wall transplantation.
